# Impact of implementation of World Health Organization National Action Plans on antibiotic rates: a time series analysis of 37 countries

**DOI:** 10.1017/ice.2025.10293

**Published:** 2025-11

**Authors:** Tate William Miner, Katherine Callaway Kim, Scott Rothenberger, Shanzeh Chaudhry, Mina Tadrous, Katie J. Suda

**Affiliations:** 1 Department of Medicine, Division of General Internal Medicine, University of Pittsburgh School of Medicine, Pittsburgh, PA, USA; 2 Leslie Dan Faculty of Pharmacy, University of Toronto, Toronto, ON, Canada; 3 Center for Healthcare Evaluation, Research, and Promotion, VA Pittsburgh Healthcare System, Pittsburgh, PA, USA

## Abstract

We conducted an interrupted time series analysis to assess changes in antibiotic sales in 37 countries that implemented National Action Plans (NAPs) between 2013 and 2018. Overall, NAP implementation was not associated with changes in antibiotic sales two years later, with country-specific effects ranging from a 38.3% decrease to 65.3% increase.

## Introduction

Antimicrobial resistance (AMR) poses a threat to global health. Without action to address contributing factors, AMR is poised to become a leading cause of mortality worldwide.^1^ Deaths associated with AMR could reach 8.2 million by 2050, up from 4.7 million in 2021.^2^ Although this increased disease burden is projected to fall disproportionately on lower and middle-income countries, AMR (and infectious diseases in general) do not respect national borders. Combating the threat of AMR requires coordination on an international scale.

In 2015, the World Health Organization (WHO) issued the Global Action Plan on AMR (GAP), an initiative aimed at reducing AMR by improving infection control, expanding stewardship, and curtailing inappropriate antimicrobial use.^3^ Critical to GAP success is the adoption of National Action Plans (NAPs) by WHO member nations. Tailored to country-specific needs, NAPs identify steps for health agencies, regulators, and nongovernmental organizations responsible for policy implementation. Despite the central importance of NAP policies, evidence of their impact on antimicrobials is limited. Two analyses assessing the impact of the COVID-19 pandemic on antibiotic trends found a small but significant decrease in sales among high-income countries, while rates remained flat or increased among lower and middle-income countries.^4,5^

To date, studies of NAP effectiveness have focused on specific countries or use the WHO GAP release date without considering country-specific implementation delays.^4,6^ Therefore, our objective was to examine post-NAP implementation changes in antibiotic utilization. We add to the literature by using international sales data and country-specific implementation dates, showing the impact of the NAP across multiple countries.

## Methods

We conducted a repeated cross-sectional study of pharmaceutical sales data from two years prior and after the implementation date of each country’s NAP. Data on antimicrobial sales from 2011–2019 were sourced from IQVIA MIDAS^®^, an IQVIA proprietary information service available on a confidential basis by subscription from IQVIA, which captures an average of 89% of wholesale retail and drug sales across 79 countries and reflects estimates of marketplace activity.^7^ Copyright IQVIA. All rights reserved. Purchase rates were reported quarterly in standardized units (1 pill/capsule/vial/5mL oral liquid), and we adjusted them per 1 million population.^7^ NAP policy documents were accessed from the WHO website.^8^

Our analysis included all countries represented in the IQVIA MIDAS dataset with NAP policies implemented from January 2013 to January 2018 (*n* = 37). We used the country-specific NAP implementation date as the index date. From this index date, we defined the pre-intervention period as 8-quarters prior and post-implementation as 8-quarters after the index date. Plans published after January 2018 were excluded to avoid confounding with COVID-19, due to volatility in antibiotic purchasing.^4^

We conducted an interrupted time series analysis using mixed effects negative binomial models with a log-link to assess level and trend changes in the antibiotic sales rate in the 8-quarters after each country’s NAP implementation, relative to 8-quarters pre-implementation. To estimate rates, we included each country’s population, as reported by the World Bank,^9^ as an offset term. We included sinusoidal terms to account for seasonality and random effects corresponding to all main effects to estimate country-specific changes in rates after 8-quarters. Our primary outcome was the relative change in the antibiotic rate 2-years post-NAP implementation, defined as the combined effect of the modeled level and trend changes at quarter 8 relative to the expected rate from the projected baseline trend assuming no intervention occurred. For certain subgroup analyses (AWaRE category, by-sector), some random effects had to be simplified to achieve model convergence.

We stratified data by sector (retail/hospital) and prescription status (prescription/non-prescription) to assess post-NAP variations. We also conducted stratified analyses by the WHO’s AWaRE system, which categorizes antibiotics based on their susceptibility to resistance (Access/Watch/Reserve).^10^

## Results

Over the study period, the quarterly antimicrobial sales rate was 4156 units per 1000 population. This rate was highest for the Access AWaRE category (2030 units per 1000 population; 48.8% of antimicrobials), followed by the Watch category (993 units per 1000 population; 23.9%) and the Reserve category (7 units per 1000 population; 0.002%). By sector, the hospital sales rate was 923 units per 1000 population (22.2%), and the retail rate was 3234 units per 1000 population (78.8%). By prescription status, the rate for prescription-bound antimicrobials was 3255 units per 1000 population (78.3%), while the non-prescription-bound rate was 314 units per 1000 population (7.6%).

**Figure 1.**
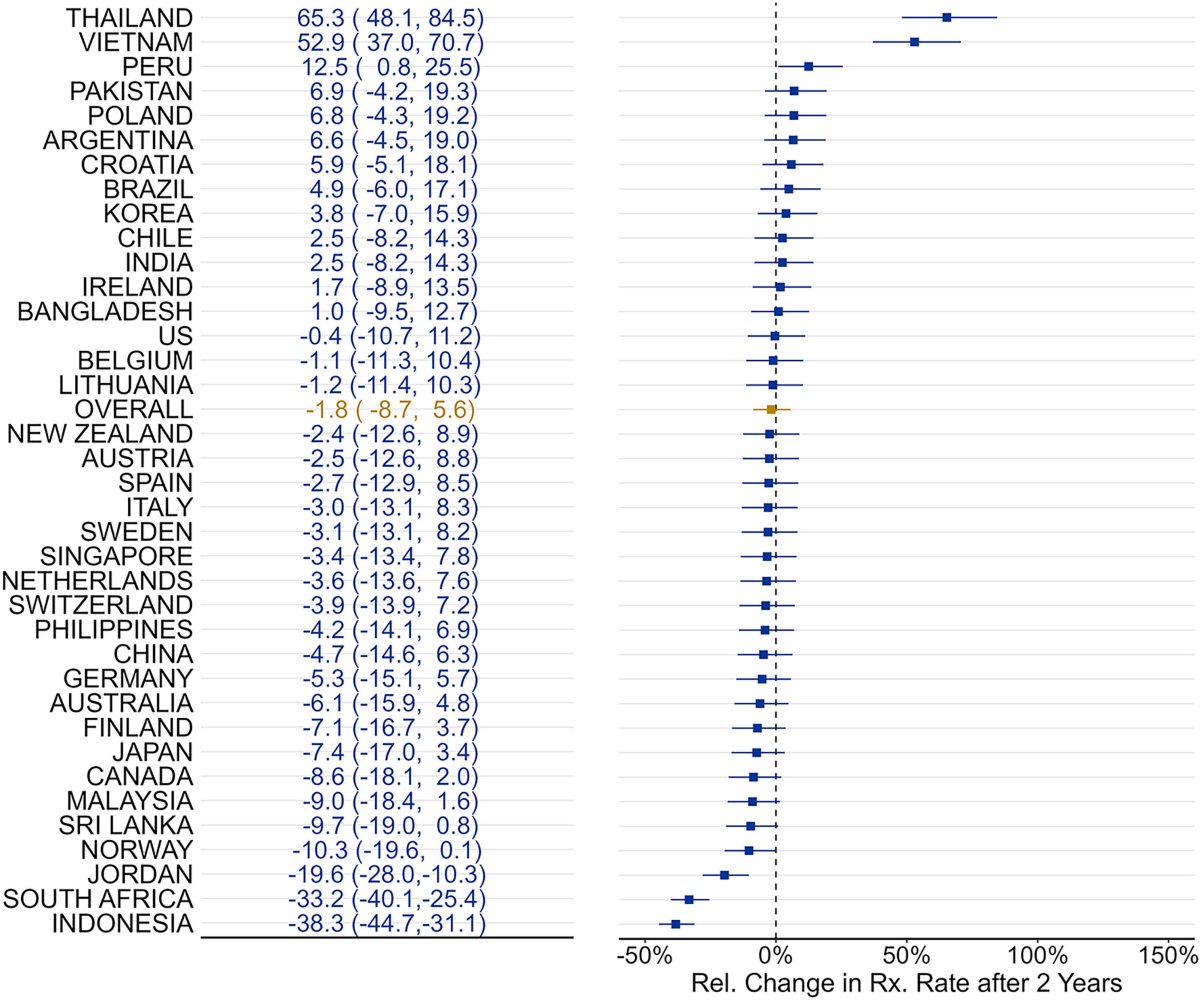
Overall changes in relative antibiotic purchases 2 years after National Action Plan implementation. *Notes*: Author analysis is based on IQVIA MIDAS^®^ quarterly volume sales data for the period Q1 2011 to Q4 2019, reflecting estimates of real-world activity. Copyright IQVIA. All rights reserved.

Across all countries, the average level change post-NAP implementation was −0.01 log units (*p* = .66), and the average trend decreased by −0.0014 log units per quarter (*p* = .73; Appendix Figure 1). Accounting for both level and trend changes, the antibiotic sales rate was 1.8% [−8.7, 5.6] lower at 8-quarters post-NAP-implementation relative to pre-period trends. Country-specific effects ranged from −38.3% to 65.30% (Figure 1). Only 3 countries experienced significant decreases (Jordan, −19.6% [−28.0, −10.3]; South Africa, −33.2% [−40.1, −25.4]; and Indonesia, −38.3% [−44.7, −31.1]), while 3 countries saw significant increases (Peru, 12.5% [0.8, 25.5]; Vietnam, 52.9% [37.0, 70.7]; and Thailand, 65.3% [48.1, 84.5]).

### Secondary analyses

Stratified by AWaRe category, sales changed by an average of −0.9% [−8.5, 7.3] units for Access, −0.9% [−8.4, 4.6] for Watch, and −12.9% [−31.9, 11.3] for Reserve drugs by 8-quarters post-implementation after accounting for level and trend changes (Table 1). Hospital and retail purchases were 4.2% [−9.2, 19.5] and 1.5% [−8.4, 12.6] higher than expected. Prescription-bound and non-prescription-bound purchases were −0.8% [−7.6, 6.6], and −6.1% [−13.7, 2.3] lower than expected.

**Table 1.** Stratified changes in antimicrobial sales volume after National Action Plan implementation

Variable	Mean purchases per capita at intervention date	Overall relative change after 8 quarters	Level change at intervention date^**^	Trend change in post-intervention period^***^
Overall	4156	−1.8% [−8.7, 5.6]	−1.78% [−4.9, 1.4]	−0.01% [−.78, .79]
**AWaRe Category** ^*^				
Access	2030	−0.9% [−8.5, 7.3]	−0.33% [−3.3, 2.6]	−0.07% [−.97, .82]
Watch	993	−2.1% [−8.4, 4.6]	2.3% [−18.4, 23.1]	−0.56% [−1.2, 1.7]
Reserve	7	−12.9% [−31.9, 11.3]	−7.6% [−31.8, 16.6]	−0.76% [−3.1, 1.7]
**Sector**				
Hospital	922	4.2% [−9.2, 19.5]	−1.1% [−8.7, 6.5]	0.65% [−.72, 2.0]
Retail	3233	1.5% [−8.4, 12.6]	0.57% [−2.4, 3.5]	0.11 [−1.1, 1.3]
**Prescription status**				
Prescription bound	3255	−0.8% [−7.6, 6.6]	−0.53% [−3.4, 2.4]	−0.02% [−1.0, .75]
Non-prescription bound	314	−6.1% [−13.7, 2.3]	−1.3% [−6.1, 3.6]	−0.60% [−1.5, .22]

* The WHO’s AWaRE categorization system classifies antibiotics based on their susceptibility to resistance (Access, Watch, Reserve). A complete list of included antibiotics can be found at https://www.who.int/publications/i/item/WHO-MHP-HPS-EML-2023.04.

** Estimated average percentage change in antimicrobial purchases immediately following the implementation date.

*** Estimated average percentage change in the rate of antimicrobial purchasing during the post-implementation period relative to the pre-implementation period.

^a^Across all countries, the average level change post-NAP implementation was −1.78% (*p* = .66), and the average sales rate decreased from pre- to post-implementation by −1.78% (*p* = 0.73).

^b^AWaRE categorization and Prescription Status were unclassified or missing for 27.3% and 14.1% of drugs, respectively.

^c^To achieve model convergence, terms were removed from the following subgroup analyses: Access – *sin and cos, time_post, quarter*; Reserve – *sin and cos*; Sector – *sin and cos*.

Notes. Author analysis is based on IQVIA MIDAS^®^ quarterly volume sales data for the period Q1 2011 to Q4 2019, reflecting estimates of real-world activity. Copyright IQVIA. All rights reserved.

## Discussion

There is little evidence that NAP implementation has been associated with reduced antimicrobials two years later. When stratified by sector, prescription status, and AWaRE categorization, the association between the NAP and sales rates remained insignificant. One explanation may be the limited scope of the NAPs regarding public-private partnerships needed for plan implementation or specific targets for antimicrobial use. Although antimicrobial optimization was a strategic objective in every policy, the plans seldom included specific commitments to improve use. Of the 26 NAPs available in English,^8^ only 7 NAPs identified antimicrobial reduction as an explicit goal. Of those 7, only 3 set a measurable target to improve antimicrobial use (Appendix Table 1). However, the extent to which NAP implementation was being monitored was unclear. These gaps between policy adoption and implementation fidelity may further hinder the impact of NAPs.

This study has limitations. First, our sample was limited to countries included in the dataset with NAPs implemented between 2013 and 2018 and therefore excluded 85 countries with published NAPs. A disproportionate number of excluded plans (*n* = 67) were from low and middle-income countries, though this overrepresentation of high-income countries is more likely to overstate NAP effectiveness.^4^ For those countries that did see a significant change, changing economic, political, or epidemiological conditions may have influenced results. Lastly, the rate of antimicrobial sales is not the only metric for measuring the effectiveness of AMR strategies – other WHO objectives, such as expanding infection control, surveillance, and research, are critical. Particularly for lower and middle-income countries, expanding access to appropriately prescribed antimicrobials is as important as limiting inappropriate use. Thus, increases in purchases are not necessarily a sign of inadequate health policy.

The international effort to contain AMR requires not only broad health policy consensus but also political commitment, actionable goals, local and national infrastructure, and the capacity to translate policy into effective action. These findings underscore the need to develop more rigorous methods of assessing NAP effectiveness and closely study the strategies of countries that realized a decrease in consumption.

## Supporting information

Miner et al. supplementary material 1 Miner et al. supplementary material

Miner et al. supplementary material 2 Miner et al. supplementary material

Miner et al. supplementary material 3 Miner et al. supplementary material

## Supplementary material

To view supplementary material for this article, please visit https://doi.org/10.1017/ice.2025.10293
