# Supplementary figures and images for: Impact of implementation of World Health Organization National Action Plans on antibiotic rates: a time series analysis of 37 countries
Source: Infect Control Hosp Epidemiol. 2025 Sep 18;46(11):1171–4. doi: 10.1017/ice.2025.10293 (PMC12620063; doi:10.1017/ice.2025.10293)

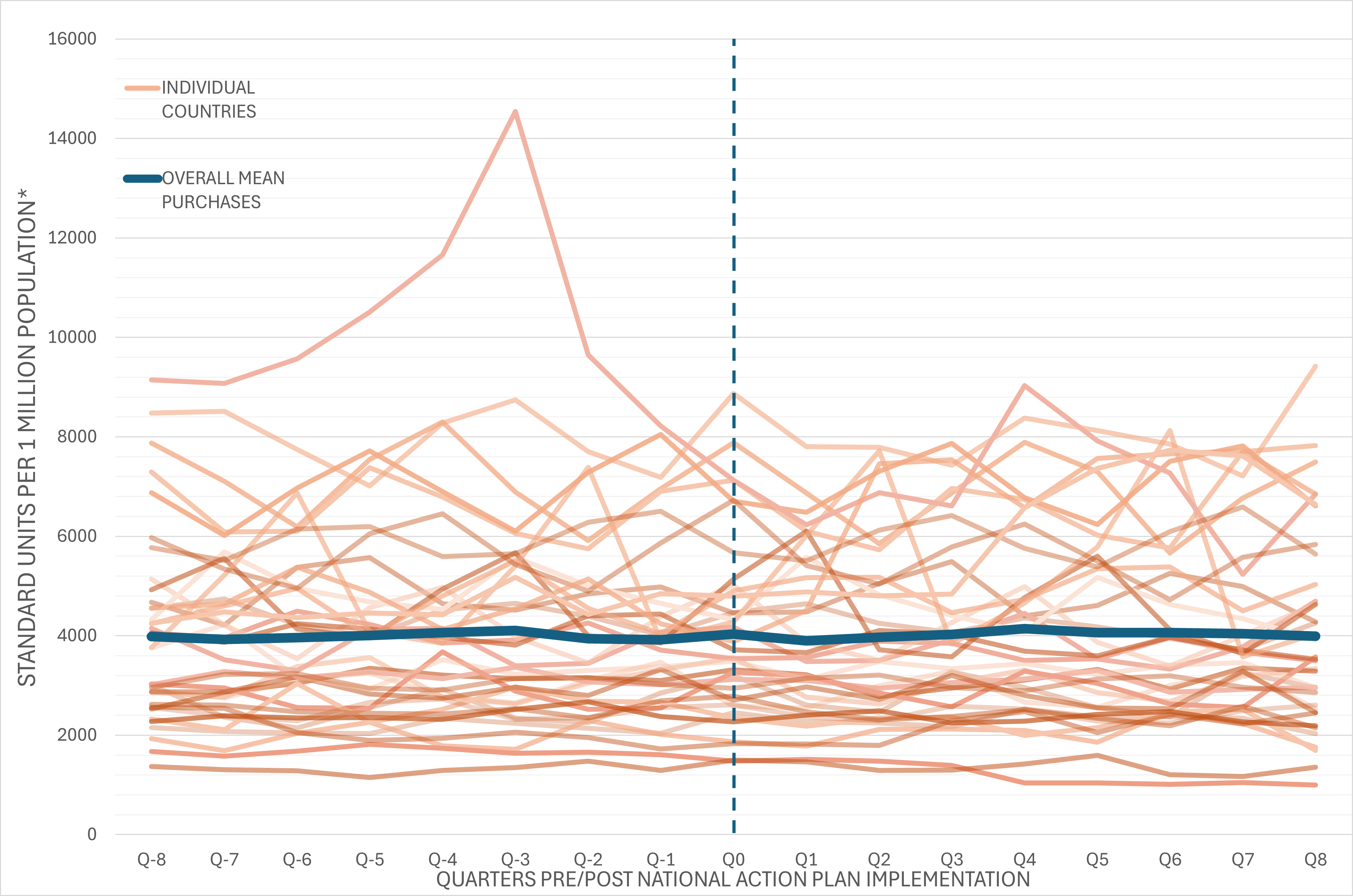

Supplement: Miner et al. supplementary material 2 — Miner et al. supplementary material [file S0899823X25102936sup002.tiff]
