# Supplementary material for: Impact of implementation of World Health Organization National Action Plans on antibiotic rates: a time series analysis of 37 countries
Source: Infect Control Hosp Epidemiol. 2025 Sep 18;46(11):1171–4. doi: 10.1017/ice.2025.10293 (PMC12620063; doi:10.1017/ice.2025.10293)
Supplement: Miner et al. supplementary material 3 — Miner et al. supplementary material [file S0899823X25102936sup003.docx]

**Appendix Figure 1. Overall trends in antimicrobial sales volume 8 quarters pre- and post-National Action Plan implementation**

**
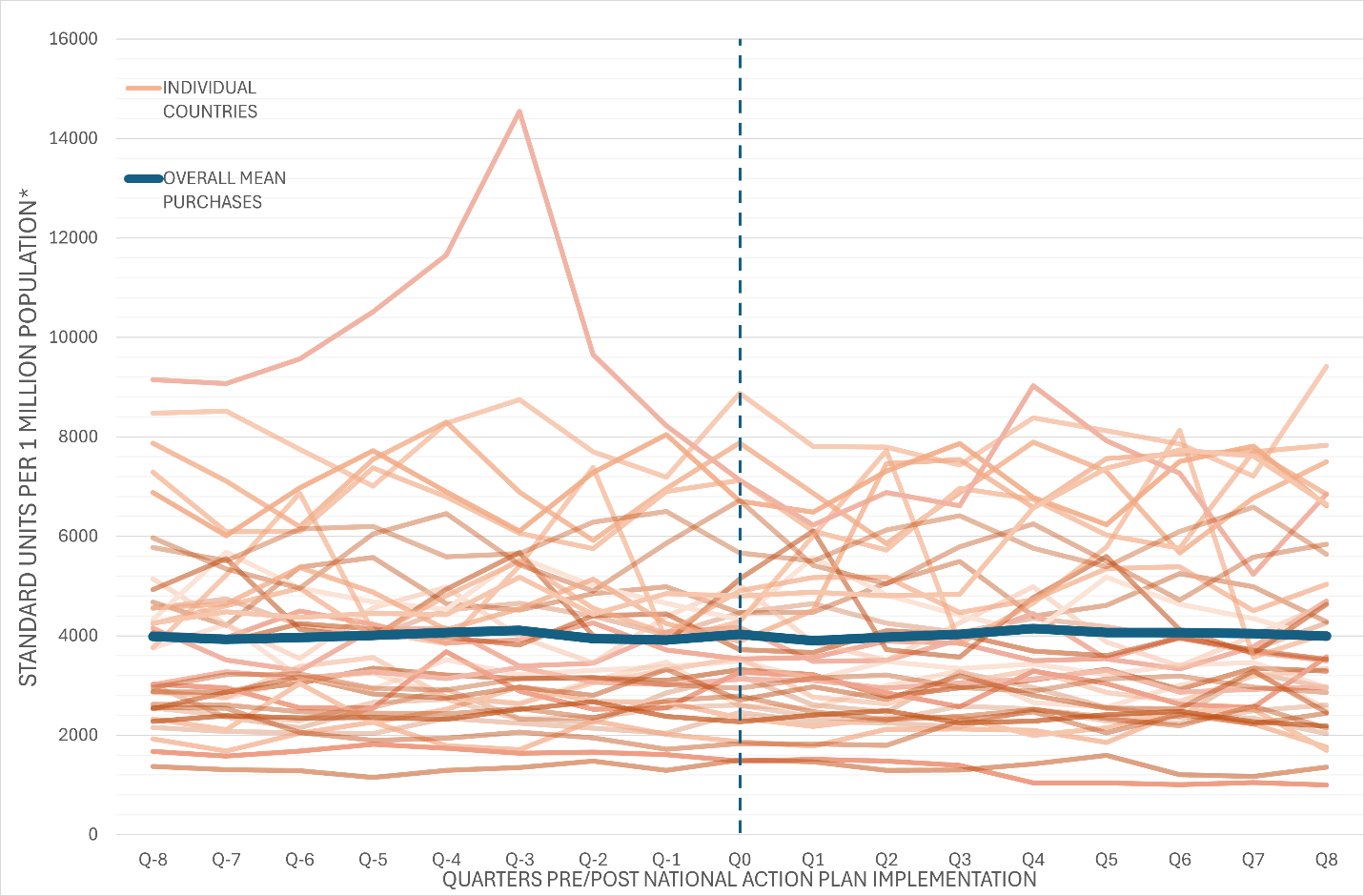
**

*1 pill/capsule/vial/5mL oral liquid

Notes:

For the overall mean purchases (solid blue line), the average level change post-NAP implementation was -0.01 log units (p=.66), and the average trend decreased by -0.0014 log units per quarter (p=.73). Accounting for both level and trend changes, the antibiotic sales rate was 1.8% [-8.7, 5.6] lower at 8-quarters post-NAP-implementation relative to pre-period trends.

Author analysis is based on IQVIA MIDAS^®^ quarterly volume sales data for the period Q1 2011 to Q4 2019, reflecting estimates of real-world activity. Copyright IQVIA. All rights reserved.
